# Cardiac biomarkers in acute respiratory distress syndrome: a systematic review and meta-analysis

**DOI:** 10.1186/s40560-021-00548-6

**Published:** 2021-04-26

**Authors:** Dilip Jayasimhan, Simon Foster, Catherina L. Chang, Robert J. Hancox

**Affiliations:** 1 Respiratory Research Unit, Department of Respiratory Medicine, Waikato Hospital, Pembroke Street, Hamilton, 3204 New Zealand; 2 Department of Preventative and Social Medicine, Otago Medical School, University of Otago, Dunedin, New Zealand

**Keywords:** Acute respiratory distress syndrome, Respiratory failure, Troponin, Brain natriuretic peptide, Critical care

## Abstract

**Background:**

Acute respiratory distress syndrome (ARDS) is a leading cause of morbidity and mortality in the intensive care unit. Biochemical markers of cardiac dysfunction are associated with high mortality in many respiratory conditions. The aim of this systematic review is to examine the link between elevated biomarkers of cardiac dysfunction in ARDS and mortality.

**Methods:**

A systematic review of MEDLINE, EMBASE, Web of Science and CENTRAL databases was performed. We included studies of adult intensive care patients with ARDS that reported the risk of death in relation to a measured biomarker of cardiac dysfunction. The primary outcome of interest was mortality up to 60 days. A random-effects model was used for pooled estimates. Funnel-plot inspection was done to evaluate publication bias; Cochrane chi-square tests and *I*^2^ tests were used to assess heterogeneity.

**Results:**

Twenty-two studies were included in the systematic review and 18 in the meta-analysis. Biomarkers of cardiac stretch included NT-ProBNP (nine studies) and BNP (six studies). Biomarkers of cardiac injury included Troponin-T (two studies), Troponin-I (one study) and High-Sensitivity-Troponin-I (three studies). Three studies assessed multiple cardiac biomarkers. High levels of NT-proBNP and BNP were associated with a higher risk of death up to 60 days (unadjusted OR 8.98; CI 4.15-19.43; *p*<0.00001). This association persisted after adjustment for age and illness severity. Biomarkers of cardiac injury were also associated with higher mortality, but this association was not statistically significant (unadjusted OR 2.21; CI 0.94-5.16; *p*= 0.07).

**Conclusion:**

Biomarkers of cardiac stretch are associated with increased mortality in ARDS.

**Supplementary Information:**

The online version contains supplementary material available at 10.1186/s40560-021-00548-6.

## Introduction

Acute respiratory distress syndrome (ARDS) is a leading cause of morbidity and mortality in the intensive care unit (ICU) [1]. A heterogeneous clinical syndrome, its definition relies on the exclusion of acute respiratory failure secondary to left heart failure or fluid overload [2]. Prognostic factors identified for this condition include age, ethnicity, comorbidities, illness severity scores, PaO2/FiO2 ratios and ventilatory parameters [3, 4]. Patients with ARDS and high baseline risk of mortality may respond differently to treatment [5]. Early identification of these patients using a biomarker may be useful in guiding clinical management.

Biomarkers of cardiac stretch such as brain natriuretic peptide (BNP) and N-terminal-probrain-natriuretic-peptide (NT-proBNP) are well established in the diagnosis and prognosis of heart failure [6]. Similarly, biomarkers of cardiac injury, such as Troponin-T and Troponin-I, are valuable in the diagnosis and prognostication of myocardial infarction [7]. These biomarkers also have prognostic value in pulmonary diseases such as pneumonia and chronic obstructive pulmonary disease [8, 9]. Whether they can be used to assess mortality risk in patients with ARDS is unknown.

The aim of this systematic review and meta-analysis is to examine the association between biomarkers of cardiac stretch or cardiac injury and short-term mortality in patients with ARDS. We hypothesised that elevated levels of these biomarkers would be associated with a higher mortality.

## Methods

We followed the Preferred Reporting Items for Systematic Reviews and Meta-Analyses (PRISMA) and Meta-analysis for Observational Studies in Epidemiology (MOOSE) statements in conducting and reporting this systematic review and meta-analysis [10]. Our protocol is registered on PROSPERO (CRD42020154072).

### Search strategy and study selection

On the 22^nd^ of June 2020, we searched the databases of PubMed, EMBASE, Web of Science and Cochrane Library using a combination of the terms ‘Cardiac biomarkers’, ‘Troponin’, ‘High Sensitivity Troponin’, ‘High Sensitivity Troponin-T’, ‘High Sensitivity Troponin-I’, ‘NT-ProBNP’, ‘N-Terminal Pro BNP’, ‘N-Terminal Pro Brain Natriuretic Peptide’, ‘Brain Natriuretic Peptide’, ‘B-Type NP’ OR ‘BNP’, ‘Acute Respiratory Distress Syndrome’, ‘ARDS’, ‘Acute Lung Injury’ and ‘ALI’. We included retrospective or prospective observational cohort studies, case-control studies and observational data drawn from randomised controlled trials that reported the mortality of ICU patients with ARDS in relation to measured cardiac biomarkers. Studies were included if ARDS was defined based on the Berlin Definition or the American European Consensus Conference definitions [2, 11]. Eligible outcome events were defined as all-cause mortality up to 60 days, in-hospital mortality, or in-ICU mortality. We excluded studies with subjects under the age of 16. We did not exclude any studies on the basis of methodological standards, sample size, duration of follow-up and publication year or language.

### Data extraction

Two reviewers (DJ and SF) screened article titles and abstracts and obtained full-text articles where eligibility was definite or unclear. Final decisions on paper inclusion were made by consensus between the two reviewers. Data were extracted from eligible articles using a predefined protocol. Individual item disagreements between the reviewers were resolved by consensus. Extracted information included first author, year of publication, year of study, number of patients at baseline, baseline characteristics including mean age, sex distribution, mean illness severity score, type of cardiac biomarker assay studied, mean or median biomarker levels in the whole sample and separately in survivors and non-survivors, follow-up duration, and the number of deaths, relative risk of death, odds of death, odds ratios (OR), relative risk ratios, hazard ratios (HR), ICU length of stay and hospital length of stay in the index and comparator groups. The primary outcome was all-cause mortality up to 60 days (including in-hospital or in-ICU mortality). Missing data were obtained by contacting corresponding authors via email. If no response was achieved on first attempt, a follow-up email was sent 2 weeks later. If there was no further response, data were considered not reported.

### Quality assessment

Risk of bias assessment was performed using the Quality in Prognosis Studies (QUIPS) tool [12]. Studies were evaluated over six domains: study participation, study attrition, prognostic factor measurement, outcome measurement, study confounding and statistical analysis and reporting. Each domain was graded as having high, moderate or low risk of bias. No summated score of the overall risk of bias for studies was assigned. Studies were tabulated and the level of evidence evaluated using the modified Grading of Recommendations Assessment, Development, and Evaluation (GRADE) framework [13].

### Statistical analysis

Meta-analyses were grouped based on biomarkers of cardiac stretch or injury and further subgrouped according to the specific biomarker. Unadjusted ORs reported in the meta-analyses were calculated from 2×2 contingency tables when possible. If the study reported an unadjusted OR, but not enough data to reconstruct a 2×2 contingency table, the OR reported in the study was used in the pooled analysis. If a study reported neither data for a contingency table nor an unadjusted OR, sensitivity and specificity reported based on receiver operating characteristics curve (ROC) analysis were used to derive data for a contingency table. Dichotomous variables were calculated using the Mantel-Haenszel statistic and outcomes are reported using a random-effects model to allow for interstudy variability. Cochrane chi-square test and the *I*^2^ test were used to assess between-study heterogeneity, with a *p* value <0.10 and *I*^2^ statistic >50% indicating significant heterogeneity respectively. Pooled ORs were reported with 95% confidence intervals and a test for overall effect using a *Z* statistic, with a *p* value <0.05 considered statistically significant. Publication bias was assessed visually using funnel plots. All analyses were performed using Review Manager (RevMan) version 5.4 (The Cochrane Collaboration, 2020).

## Results

**Fig. 1:**
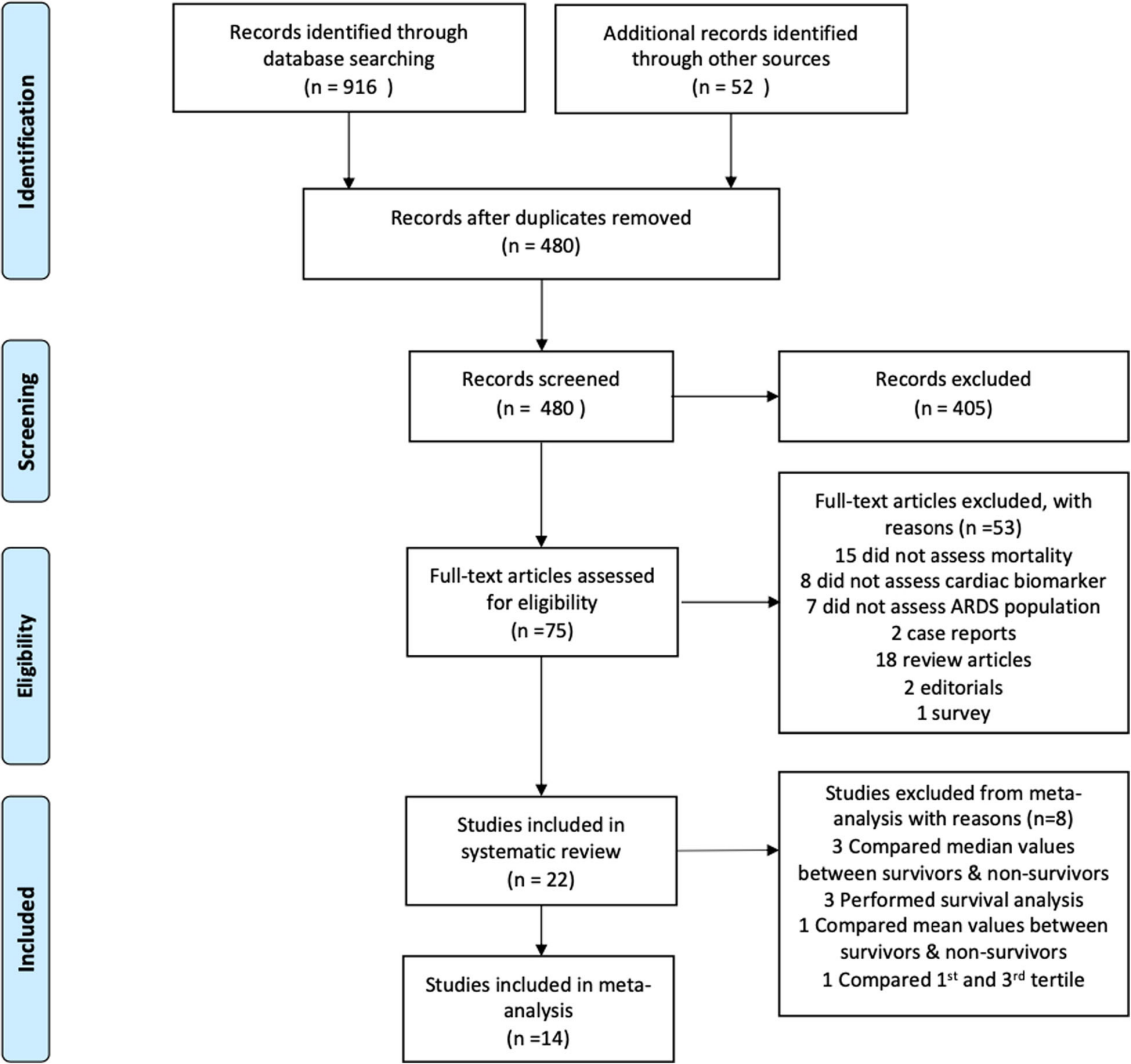
PRISMA diagram

The search strategy identified 968 records. Four hundred eighty-eight were duplicates leaving 480 unique items for screening. After screening titles and abstracts, 75 full texts were reviewed. Fifty-three articles were excluded for reasons stated in the PRISMA diagram (Fig. 1) and 22 studies were included in the systematic review. Fourteen were included in the meta-analysis.

### Systematic review

Most studies (18) were prospective cohort studies with two large retrospective analyses of randomised controlled trials (Table 1). The prognostic utility of NT-ProBNP was assessed by nine studies [14–22], BNP by six [23–28], Troponin-T by two [29, 30], Troponin-I by one [31], high-sensitivity-Troponin-I by one [32], and multiple cardiac biomarkers by three studies [33–35].

**Table 1:** Description of all studies included

Study	Cardiac biomarker	Type of study	No patients	Mean age	Baseline illness severity score	Mortality outcome	Overall mortality rate (%)	Biomarker cut-point (method determined)	Unadjusted results	Adjusted results	Adjusted variables
Bajwa 2008 [14]	NT-proBNP	Prospective cohort	177	60.1	APACHE III: 79.6	60 day	40	6813 pg/ml (ROC)	OR: 4.24 (CI 2.17-8.27)	OR: 2.36 (CI 1.11-4.99)	Age, APACHE III
Park 2011 [15]	NT-proBNP	Prospective cohort	49	64	APACHE II: 22 SOFA: 8	28 day	63.26	NA	Median value in survivors vs non-survivors (3999 vs 2819, *p*=0.719)	NA	NA
Lai 2017 [16]	NT-proBNP	Prospective cohort	61	65	APACHE II: 23 SOFA: 11	28 day	55.7	NA	HR: 1.006 (1.002–1.010) *p*=0.002	HR: 1.009 (CI 1.004–1.013)	Age, APACHE II
Lin 2012 [17]	NT-proBNP	Prospective cohort	87	61	APACHE II: 20	30 day	31	2417 pg/ml (ROC)	HR: 2.52 (1.58-3.67) *p*=0.021	2.18 (1.54-4.46) *p*=0.026	APACHE II, LIS Cr, copeptin, increase in copeptin, increase in BNP
Zhou 2015 [18]	NT-proBNP	Prospective cohort	87	47.1	APACHE II: 22.78	28 day	32.2	333.92 pg/ml (ROC)	Median value in survivors vs non-survivors (231.59 vs299.98, *p*<0.05)	HR: 1.925 (CI 1.512-3.026) *p*<0.05	APACHE II, Murray score, extrapulmonary organ failures, oxygenation index
Ji 2016 [19]	NT-proBNP	Prospective cohort	80	NR	NR	28 day	37.5	NA	Median value in survivors vs non-survivors (3414 vs 5212, *p*<0.01)	NA	NA
Xu 2013 [20]	NT-proBNP	Prospective cohort	50	49.0	NR	28 day	20	335 pg/ml (ROC)	ROC AUC 0.96 Sn 80%, Sp 92.5% Median value in survivors vs non-survivors (237.64 vs 287.86, *p*<0.01)	NA	NA
Su 2018 [21]	NT-proBNP	Prospective cohort	51	49.3	APACHE II: 22.86	28 day	47.6	NR	ROC AUC 0.832 Sn 79.2%, Sp 74.1% Median value in survivors vs non-survivors (2868 vs 3881, *p*<0.05)	NA	NA
Ferris 2019 [22]	NT-proBNP	Retrospective analysis of RCT	262	55.7	SOFA: 8.54	28 day	27	751.553 pg/ml (median)	OR: 1.93 (CI 1.27-2.92), patients randomised to simvastatin with high BNP had lower mortality than placebo.	NA	NA
Bonizzoli 2018 [23]	NT-proBNP Trop-I	Prospective cohort	30	58	SAPS II: 43.2	ICU	33.3	NA	Median value in survivors vs non-survivors (NT-proBNP 1250 vs 3091 *p*= 0.009, Trop-I 0.12 vs 0.32 *p*= 0.04)	NA	NA
Nassar 2010 [24]	NT-proBNP Trop-I TropT	Prospective cohort	20	58.9	NR	30 day	65	NT-proBNP: 1200 pg/mL, Tn-I 1.5 ng/mL, Tn-T 0.5 ng/mL.	Median value in survivors vs non-survivors (NT-proBNP 712.0 vs 8975.9, Trop-I 0.2 vs 4.75, Trop-T 0 vs 0.51 *p*<0.001 for all three biomarkers), NT-proBNP sn 92%, sp 100%, Trop-I sn 100%, sp 57%, Trop-T sn 100%, sp 43%	NA	NA
Sun 2015 [25]	BNP	Prospective cohort	59	58.4	NR	28 day	30.5	NA	Mean value in survivors vs non-survivors (128.99 SD45.2 vs 267.07 SD 45.06, *p* <0.01)	NA	NA
Semler 2016 [26]	BNP	Retrospective analysis of RCT	625	49.4	APACHE III: 93.25	60 day	21.76	825 pg/ml (median)	OR: 0.927 (CI 0.811-1.062)	NA	NA
Cepkova 2011 [27]	BNP	Prospective cohort	42	62	SAPS II: 45 APACHE II: 21	30 day	36	NA	Median value in survivors vs non-survivors (385 vs 420 pg/ml, *p*=0.71)	NA	NA
Karmpaliotis 2007 [28]	BNP	Prospective cohort	51	62	APACHE II: 20	In-hospital mortality	42.4	NA	Mortality in lowest tertile vs highest tertile BNP (26.1% vs 60.9% *p*=0.07)	NA	NA
Rhee 2007 [29]	BNP	Prospective cohort	47	65.7	APACHE II: 21	30 day	63.8	585 pg/ml	Median value in survivors vs non-survivors (219.5 vs 492.3 pg/ml *p*=0.013) Sn 43% Sp 94%	NA	NA
Lin 2010 [30]	BNP	Prospective cohort	86	NR	NR	14 day	63.9	329.5 pg/ml (ROC)	Median value in survivors vs non-survivors (179.5 vs 550.8 pg/ml *p*<0.01) ROC AUC 0.96 Sn 80%, Sp 92.5% at cut-off 335 pg/ml	NA	NA
Rivara 2012 [31]	Trop-T	Prospective cohort	177	62.1	APACHE III: 79.72	60 day	39.5	0.036 ng/mL (ROC)	HR 1.33 (CI 1.10–1.62) [*p*=0.003] Median in survivors vs non-survivors (0.022 vs 0.042 *p*=0.008)	HR: 1.44 (CI 1.14-1.81) [*p*=0.002]	Daily MODS, VFDs
Bajwa 2007 [32]	Trop-T	Prospective cohort	248	61.5	APACHE III: 82.4	60 day	47	0.09 ng/mL (PD)	OR: 4 1.72 (CI 1.02-2.90) [*p*=0.04] ROC AUC Trop-T 0.63	OR: 1.48 (CI 0.82-2.69) [*p*=0.19]	APACHE III, presence of septic shock, Cr, diabetes
Lazzeri 2016 [33]	Trop-I	Prospective cohort	42	55.5	SAPS II: 39.7	ICU	47.6	<0.1 ng/ml (PD)	Mortality in high vs low Trop-I (56.25% vs 42.3% *p*= NS)	NA	NA
Austin 2009 [34]	Trop-I CKMBI	Retrospective cohort	51	62.3	NR	ICU	43	Tn-I: 0.59ng/mL CKMBI: 5 (PD)	Mortality in high vs low Trop-I (42.8% vs 43.3% *p*=0.973)	NA	NA
Metkus 2017 [35]	HS-Trop-I	Retrospective analysis of RCT	1057	50.4	SOFA: 7.7	60 day	28	NA	HR: 1.57 (1.17-2.11) [0.003] comparing 1st with 5th quintile	HR: 1.01 (CI 0.73-1.39) [*p*=0.94]	Age, sex, randomised trial assignment (for unadjusted), further adjusted for SOFA, vasopressors, heart rate

*NT-proBNP* N-terminal probrain natriuretic peptide, *Trop-T* Troponin-T, *Trop-I* Troponin-I, *BNP* Brain natriuretic peptide, *CKMBI* Creatine kinase MB index, *HS-Trop-I* High sensitivity Troponin I, *RCT* Randomised controlled trial, *APACHE* Acute Physiology and Chronic Health Evaluation, *SOFA* Sequential Organ Failure Assessment, *NR* Not reported, *SAPS* Simplified acute physiology score, *ROC* Receiver operating characteristics curve, *OR* Odds ratio, *HR* Hazard ratio, *AUC* Area under the curve, *SD* Standard deviations, *Sn* Sensitivity, *Sp* Specificity, *MODS* Multi-organ dysfunction syndrome, *VFDs* Ventilator free days

All 22 studies provided unadjusted estimates and seven studies provided adjusted estimates of mortality risk associated with biomarkers. These estimates were reported in a variety of ways: mainly as median levels of biomarkers in survivors versus non-survivors. Other reports of effect estimates included sensitivity and specificity based on optimal cut-points from ROCs, ORs or HRs comparing patients with a high cardiac biomarker against those with a low cardiac biomarker.

Most studies had a low to moderate risk of bias in the domains of study participation, attrition and confounding (Table S1). Two studies had a moderate risk of bias in the study participation domain due to high numbers of eligible patients excluded from participation following screening: a large proportion of patients in one study did not have biomarkers measured [25]. The other study did not explain why only 60% of patients were included [26].

Two studies lacked information on follow-up duration [22, 34]. Several studies had a moderate or high risk of bias for confounding because results were not adjusted for potential confounding variables associated with prognosis such as illness severity scores. All other studies reported follow-up from 14 up to 60 days.

### Biomarkers of cardiac stretch

All 11 cohorts studying NT-proBNP found statistically significant associations between high NT-proBNP levels and mortality in univariate analyses. Only 4 of these studies reported adjusted outcomes. In all four studies, NT-ProBNP remained an independent prognostic marker for mortality in ARDS following adjustment. The most commonly adjusted factors were illness severity scores and age, followed by other factors found to be prognostic following univariate analyses in their respective studies. These factors included sex, cardiac ejection fraction, serum creatinine and copeptin, lung injury score and magnitude in the change of NT-ProBNP levels over serial measurements.

Assessment based on the GRADE tool (Table 2) suggests an overall quality of evidence that is moderate for NT-proBNP, when taking into account the limitations of imprecision, inconsistency, and publication bias, as well as the added strength of a moderate to large effect size.

**Table 2:** Grade table

Cardiac biomarker	No. of participants	No. of cohorts	Univariate			Multivariate			Phase	Limitations	Inconsistency	Indirectness	Imprecision	Publication bias	Moderate/large effect size	Dose effect	Overall quality
			+	0	−	+	0	−									
NT-proBNP	954	11	10	1	0	4	0	0	1	Yes	Yes	No	Yes	Yes	Yes	No	+++
BNP	824	6	3	3	0	0	0	0	1	Yes	Yes	No	Yes	Yes	Yes	No	+++
HS-Trop-I	1057	1	1	0	0	0	1	0	1	Yes	No	No	No	Yes	No	No	++
Trop-I	143	4	2	2	0	0	0	0	1	Yes	No	No	Yes	Yes	No	No	++
Trop-T	445	3	3	0	0	1	1	0	1	Yes	Yes	No	Yes	Yes	No	No	+

Phase, phase of investigation: Phase 1, outcome prediction research or explanatory research aimed to identify associations between potential prognostic factors and the outcome; phase 2, explanatory research aimed to confirm independent associations between potential prognostic factor and the outcome; phase 3, explanatory research aimed to understand prognostic pathways. For uni- and multivariate analyses: +, number of significant effects with a positive value; 0, number of non-significant effects; −, number of significant effects with a negative value. For GRADE factors: For overall quality of evidence: +, very low; ++, low; +++, moderate; ++++, high

Six cohorts containing 824 patients were included in our systematic review of the relationship between BNP and mortality. The largest cohort included 625 patients from a retrospective analysis of the FACTT trial and found a non-significant difference in mortality between the high BNP group and low BNP group [25]. Of the six cohorts, three showed a positive relationship between BNP levels and mortality from univariate analysis, whilst the three others showed no association. None of the studies performed multivariate analysis controlling for common confounding factors. Overall the quality of evidence in studies assessing the association between BNP and mortality was moderate based on the GRADE assessment.

### Biomarkers of cardiac injury

Our systematic review included four small cohorts with 143 patients assessing Troponin-I. Two found a positive relationship between Troponin-I and mortality on univariate analysis but not multivariate analysis. A retrospective analysis of the FACTT trial by Metkus et al. [34] was the only study comparing survival between patients with elevated and non-elevated High-Sensitivity-Troponin-I levels. It showed a significantly higher survival in the first quintile compared to the fifth quintile through univariate analysis (HR: 1.57; CI 1.17-2.11; *p* value=0.003); however, when controlled for age, sex, trial assignment, SOFA score, vasopressor requirement and heart rate, this association was not significant (HR 1.01; CI 0.73-1.39; *p*=0.94).

Three studies assessed the relationship between Troponin-T and mortality, showing an association between high Troponin-T levels and mortality from univariate analysis. Of these, only two performed a multivariate analysis with Bajwa et al. [31] showing no independent association following adjustment for Acute Physiology and Chronic Health Evaluation (APACHE) III, presence of septic shock, blood creatinine and diabetes (OR 1.48; CI 0.82-2.69; *p*=0.19). Rivara et al. [30], however, found an independent association between Troponin-T and mortality following adjustment for age, APACHE III, hepatic failure, presence of coronary artery disease, bilirubin, blood urea and lowest mean arterial pressure (HR 1.44; CI 1.14-1.81; *p*=0.002). Overall, the quality of evidence was considered low for Troponin-I and HS-Troponin-I, and very low for Troponin-T based on the GRADE tool.

### Meta-analysis

**Fig. 2:**
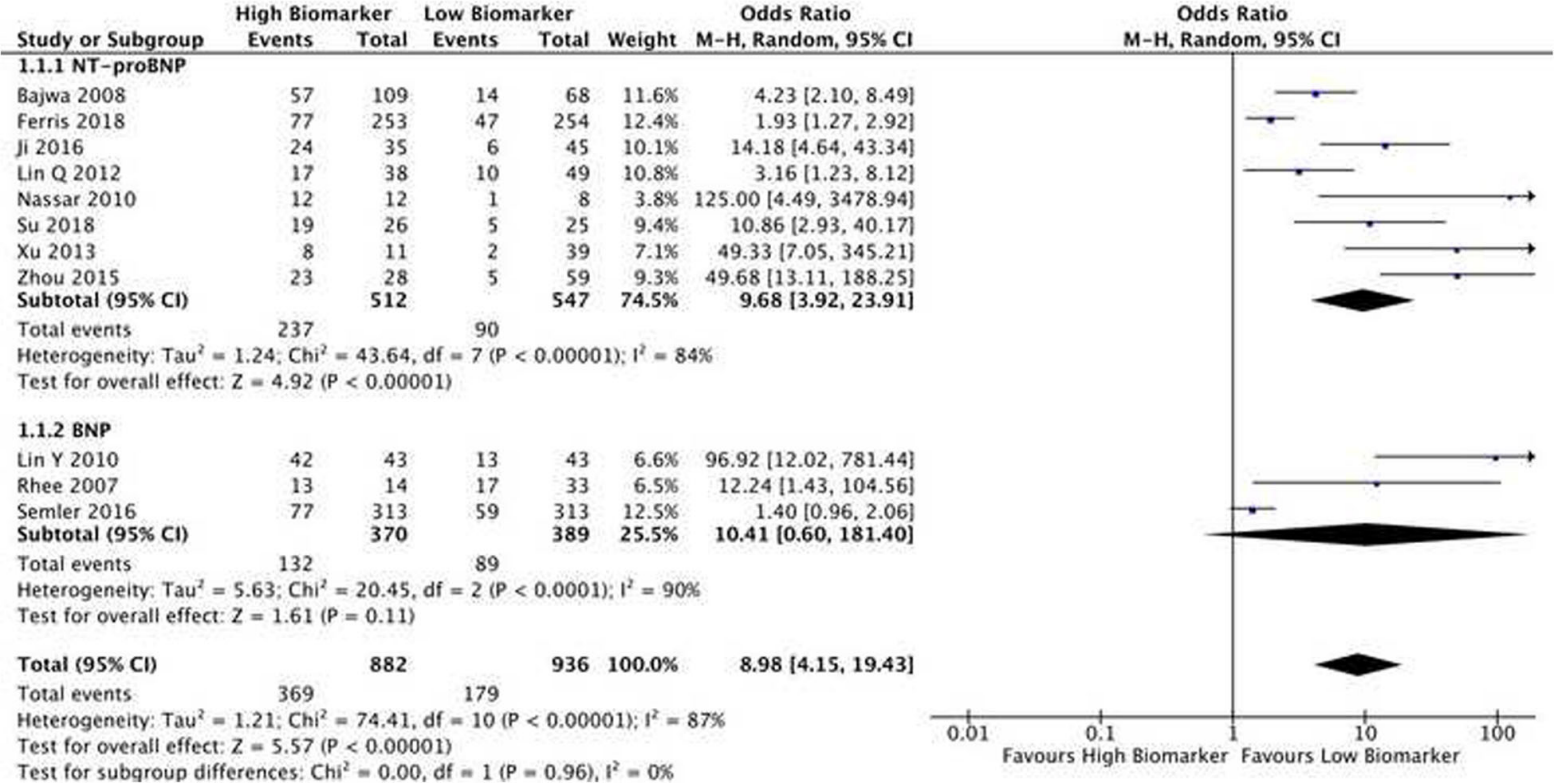
Biomarkers of cardiac stretch and mortality forest plot

We included 11 studies with 1731 patients in our meta-analysis of brain natriuretic peptides (Fig. 2). Eight studies assessed NT-proBNP and three assessed BNP. A total of 369 of 882 patients (41.8%) with a high biomarker died compared to 179 of 936 patients (20.2%) with a low biomarker, giving an unadjusted OR for mortality of 8.98 (CI 4.15-19.43; *p*<0.00001). There was significant heterogeneity between the studies in the meta-analysis (*I*^2^= 87%; *p*<0.00001), which persisted despite subgroup analyses for NT-proBNP (*I*^2^<84%; *p*<0.00001) and BNP (*I*^2^= 90%; *p*=0.0001). Subgroup analysis showed that high levels of both NT-proBNP and BNP were associated with higher mortality, although this was not statistically significant for BNP (Fig. 2). Most studies included in the meta-analysis used cut-points determined by ROC curve analysis. Despite using similar assays, these cut-points varied substantially. We conducted a sensitivity analysis using a fixed-effects model. This showed a smaller effect size, although it remained statistically significant (OR 3.16; CI 2.55-3.92; *p*<0.00001) (Figure S1).

**Fig. 3:**
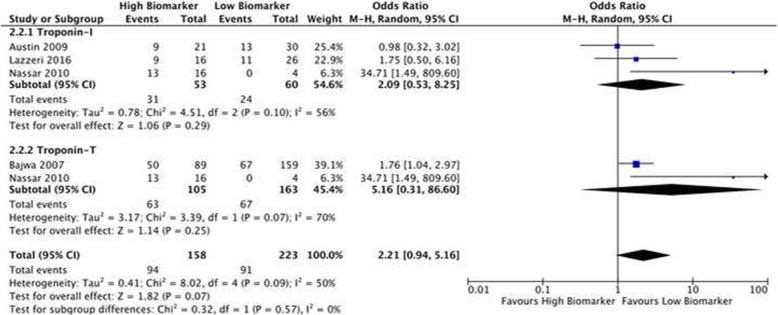
Biomarkers of cardiac injury and mortality forest plot

Five studies of cardiac troponins were included in our meta-analysis with three assessing Troponin-I and two assessing Troponin-T. High levels of troponins were associated with a non-significantly higher mortality (OR 2.21; CI 0.94-5.16; *p*= 0.07) (Fig. 3). There was significant heterogeneity between the studies (*I*^2^= 50%; *p*= 0.07). Differences in mortality between high and low biomarkers were not statistically significant for subgroups reporting either Troponin-I (OR 2.09; CI 0.53-8.25; *p*=0.29) or Troponin-T (OR 5.1; CI 0.31-86.60; *p*=0.25).

Visual assessment of funnel plots for both brain natriuretic peptides, as well as cardiac troponins, demonstrated asymmetry suggesting publication bias (Figures S2 and S3).

## Discussion

This systematic review and meta-analysis found that elevated blood brain natriuretic peptide levels are associated with higher mortality in patients with ARDS in ICU. This association is independent of illness severity score and age. These observations suggest that cardiac stretch may be a useful indicator of prognosis in ARDS.

There was also an association between cardiac troponins and mortality, but this was not statistically significant on meta-analysis perhaps due to smaller sample sizes. Thus, it remains uncertain whether cardiac injury is a predictor of prognosis in ARDS.

Our findings are consistent with a meta-analysis by Ni et al., which found that NT-proBNP had a moderate value in predicting mortality in patients with ARDS [36]. Their study differed from ours in its inclusion criteria and methods of analysis. Ni et al. generated a composite sensitivity and specificity of NT-proBNP at predicting mortality, whereas we pooled unadjusted ORs. We were able to include more studies of NT-proBNP in our meta-analysis, increasing the precision of the estimate of the findings.

The mechanism for the association between biomarkers of cardiac dysfunction and mortality in patients with ARDS is not clear. High levels of natriuretic peptides could reflect right ventricular (RV) dysfunction, a common complication of ARDS. Evidence of RV dysfunction on echocardiogram or indicated by a high transpulmonary pressure gradient in patients with ARDS are associated with higher mortality [37]. Biomarkers of cardiac stretch reflect acute RV dysfunction in many other conditions that increase RV afterload such as acute pulmonary embolism [38], and RV dysfunction is associated with poorer survival in these conditions [22]. Troponin-I may also be a marker of pulmonary hypertension and RV systolic dysfunction based on one of the studies included in our review [32].

It is plausible that there is a direct causal link between cardiac involvement and death from ARDS, such that severe ARDS leads to cardiac dysfunction or injury through a number of potential mechanisms (systemic inflammation, hypoxaemia, changes in pulmonary artery pressure) leading to an increased risk of death. Alternatively, elevated cardiac biomarkers may merely act as a proxy for the severity of ARDS. Previous human and animal studies have shown an increase in BNP levels in response to hypoxic pulmonary vasoconstriction [39–41]. BNP is also thought to have a pulmonary vasorelaxant effect that may be part of the natural compensatory response.

Moreover, elevated biomarkers of cardiac stretch may reflect systemic illness severity or presence of pre-existing comorbidities given their prognostic value in many extra-cardiac conditions such as pneumonia, sepsis, stroke and trauma [42–44]. Extrapulmonary organ injury has been shown to correlate with ARDS severity and be predictive of mortality [34, 45]. Complicating conditions such as new onset atrial fibrillation and acute kidney injury are associated with elevations in cardiac biomarkers [46, 47]. These complications in ARDS are likely to have prognostic implications and the relationship between cardiac biomarker elevation and death in ARDS may be indicative of this.

Furthermore, based on the current Berlin definition, the diagnosis of ARDS relies on clinical assessment to rule out left atrial hypertension. Demonstration of a low pulmonary artery wedge pressure via invasive measurement is no longer a pre-requisite in diagnosing ARDS [2]. Hence, it is possible that subclinical left heart failure may be under-recognised in this population and cardiac biomarkers may be a reflection of this.

This is the first systematic review and meta-analysis to examine the prognostic value of biomarkers of both myocardial stretch (brain natriuretic peptides) and cardiac injury (cardiac troponins) in patients with ARDS. There are a number of important limitations to the study. Firstly, there is considerable heterogeneity between the studies in the meta-analyses. These heterogeneities persisted in subgroup analyses. There was also evidence of publication bias and the influence of some of the smaller studies may have inflated the OR associated with high levels of BNP and NT-proBNP. We therefore performed a sensitivity analysis using a fixed-effects model (Figure S1), which weights the findings according to sample size. In this analysis, the association with higher mortality persisted, albeit at a somewhat lower OR (OR 3.16; CI 2.55-3.92). Secondly, most studies included retrospectively determined cut-points either through the use of median values or by ROC analysis. These cut-points have not been prospectively validated and varied between studies. Thirdly, the large confidence intervals, especially for studies assessing BNP and Troponin-T, reflect the lack of precision in the findings and could lead to a failure to identify statistically significant clinically important associations. The funnel plots also suggest some publication bias. Finally, several studies included in our review did not contain sufficient information to be included in the meta-analysis, which could introduce bias to our findings. However, the findings of our systematic review are consistent with our meta-analysis.

Our findings raise a number of important questions. A key gap in knowledge is an explanatory mechanism behind the association between cardiac biomarkers, particularly brain natriuretic peptides, and mortality in patients with ARDS. If this is a direct causal association, treating cardiac stretch could plausibly reduce mortality. If these biomarkers are simply indicators of severity, cardiac treatment may not help, but the biomarkers may be useful in identifying patients with a poor prognosis. However, without prospectively well-validated cut-points, the clinical utility of abnormal cardiac biomarkers in ARDS remains limited.

## Conclusion

High levels of brain natriuretic peptides, indicating cardiac stretch, are associated with a higher risk of death in patients with ARDS independently of other commonly used prognostic indicators. Further studies are required to determine if a similar relationship exists between cardiac troponins and mortality in ARDS.

## Supplementary Information

**Additional file 1: Table S1**: QUIPS Table. **Figure S1**: Sensitivity Analysis of Biomarkers of Cardiac Stretch (Fixed Effects Model). **Figure S2**: Biomarkers of Cardiac Stretch Funnel Plot. **Figure S3**: Biomarkers of Cardiac Injury Funnel Plot

## Data Availability

The datasets used and/or analysed during the current study are available from the corresponding author on reasonable request.

## Abbreviations

ARDS: Acute respiratory distress syndrome
ICU: Intensive care unit
BNP: Brain natriuretic peptide
NT-proBNP: N-terminal probrain natriuretic peptide
PRISMA: Preferred reporting items for systematic reviews and meta-analyses
MOOSE: Meta-analysis for observational studies in epidemiology
OR: Odds ratio
HR: Hazard ratio
QUIPS: Quality in prognostic studies
GRADE: Grading of recommendations assessment, development, and evaluation
ROC: Receiver operating characteristics curve
APACHE: Acute physiology and chronic health evaluation
RV: Right ventricle
