# Supplementary material for: Cardiac biomarkers in acute respiratory distress syndrome: a systematic review and meta-analysis
Source: J Intensive Care. 2021 Apr 26;9:36. doi: 10.1186/s40560-021-00548-6 (PMC8072305; doi:10.1186/s40560-021-00548-6)
Supplement: Supplementary file 1 — Additional file 1: Table S1: QUIPS Table. Figure S1: Sensitivity Analysis of Biomarkers of Cardiac Stretch (Fixed Effects Model). Figure S2: Biomarkers of Cardiac Stretch Funnel Plot. Figure S3: Biomarkers of Cardiac Injury Funnel Plot [file 40560_2021_548_MOESM1_ESM.docx]

Table S1: QUIPS Table

| Study | Participation | Attrition | Biomarker measurement | Outcome measurement | Confounding | Statistical analysis |
| --- | --- | --- | --- | --- | --- | --- |
| Bajwa 2008 | Low | Low | Low | Low | Low | Low |
| Park 2011 | Low | Low | Low | Low | Low | Low |
| Lai 2017 | Low | Low | Low | Low | Low | Low |
| Lin 2012 | Low | Low | Low | Low | Low | Low |
| Zhou 2015 | Low | Low | Low | Low | Moderate | Low |
| Ji 2016 | Low | Low | Low | Low | Moderate | Low |
| Xu 2013 | Low | Low | Low | Low | Moderate | Low |
| Su 2018 | Low | Low | Low | Low | Moderate | Low |
| Ferris 2018 | Low | Low | Low | Low | Moderate | Low |
| Bonizzoli 2018 | Low | Low | Low | Moderate | Moderate | Low |
| Nassar 2010 | Low | Low | Low | Low | Moderate | Low |
| Sun 2015 | Low | Low | Low | Low | High | Low |
| Semler 2016 | Moderate | Low | Low | Low | Low | Low |
| Cepkova 2012 | Moderate | Low | Low | Low | Low | Low |
| Karmpaliotis 2007 | Low | Low | Low | Low | Low | Low |
| Chin 2007 | Low | Low | Low | Low | Moderate | Low |
| Lin 2010 | Low | Low | Low | Low | Moderate | Low |
| Lazzeri 2016 | Low | Low | Low | Moderate | Low | Low |
| Austin 2009 | Low | Low | Low | Low | Moderate | Low |
| Rivara 2012 | Low | Low | Low | Low | Low | Low |
| Bajwa 2007 | Low | Low | Low | Low | Low | Low |
| Metkus 2017 | Low | Low | Low | Low | Low | Low |

Figure S1: Sensitivity Analysis of Biomarkers of Cardiac Stretch (Fixed Effects Model)

Figure S2: Biomarkers of Cardiac Stretch Funnel Plot

Figure S3: Biomarkers of Cardiac Injury Funnel Plot
